# A Novel Mechanism Underlying Multi-walled Carbon Nanotube-Triggered Tomato Lateral Root Formation: the Involvement of Nitric Oxide

**DOI:** 10.1186/s11671-020-3276-4

**Published:** 2020-02-26

**Authors:** Zeyu Cao, Heng Zhou, Lingshuai Kong, Longna Li, Rong Wang, Wenbiao Shen

**Affiliations:** 1 College of Life Sciences, Laboratory Center of Life Sciences, Nanjing Agricultural University, Nanjing, 210095 China; 2 Institute of Botany, Jiangsu Province and Chinese Academy of Sciences, Nanjing, 210014 China

**Keywords:** Multi-walled carbon nanotubes, Lateral root, Nitric oxide, Nitric reductase, Tomato

## Abstract

Abundant studies revealed that multi-walled carbon nanotubes (MWCNTs) are toxic to plants. However, whether or how MWCNTs influence lateral root (LR) formation, which is an important component of the adaptability of the root system to various environmental cues, remains controversial. In this report, we found that MWCNTs could enter into tomato seedling roots. The administration with MWCNTs promoted tomato LR formation in an approximately dose-dependent fashion. Endogenous nitric oxide (NO) production was triggered by MWCNTs, confirmed by Greiss reagent method, electron paramagnetic resonance (EPR), and laser scanning confocal microscopy (LSCM), together with the scavenger of NO. A cause-effect relationship exists between MWCNTs and NO in the induction of LR development, since MWCNT-triggered NO synthesis and LR formation were obviously blocked by the removal of endogenous NO with its scavenger. The activity of NO generating enzyme nitrate reductase (NR) was increased in response to MWCNTs. Tungstate inhibition of NR not only impaired NO production, but also abolished LR formation triggered by MWCNTs. The addition of *N*^G^-nitro-l-arginine methyl ester (l-NAME), an inhibitor of mammalian nitric oxide synthase (NOS)-like enzyme, failed to influence LR formation. Collectively, we proposed that NO might act as a downstream signaling molecule in MWCNT control of LR development, at least partially via NR.

## Introduction

There have many biological and biomedical applications of carbon nanotubes [1, 2]. Due to the unique ability to easily penetrate cell membranes, the biosafety of carbon nanotubes is always a debate topic [3, 4]. Meanwhile, since the production and use of carbon nanotubes grow rapidly, it becomes important to characterize the detailed mechanisms of its cytotoxicity in human beings and mammalians, and recently in plants [3–9]. It is well-known that plants and their communities are very important for humans and environment, and they are at risk of carbon nanotubes exposure either, due to buildup in soils through biosolid fertilizer application [6, 10, 11]. As the important members of carbon nanotubes, the toxicity of multi-walled carbon nanotubes (MWCNTs), consisting of multiple rolled layers of graphene, has been widely investigated. Studies in mammalian revealed that the exposure with both MWCNTs and single-walled carbon nanotube induced oxidative damage and NF-κB activation in human keratinocytes and A549 cells [9, 12]. MWCNTs and single-walled carbon nanotube can fuse with the plasma membrane, thus causing cell damage through lipid peroxidation and oxidative stress [9, 11, 13, 14]. Cytotoxicity and oxidative stress triggered by MWCNTs, as well as modest inflammatory responses, were observed in human umbilical vein endothelial cells [15]. Previous study suggested that the primary toxicity of MWCNTs in red spinach was mainly derived from reactive oxygen species (ROS) overproduction, and the toxic effects could be reversed by the supplemented ascorbic acid [7]. In this sense, MWCNTs is considered as a new stressed factor to organisms, either in animals or in plants.

Lateral root (LR) formation, an important determinant of root architecture, has been considered as an indicator of adaptive response to various stresses [16]. In higher plants, the formation of LR is influenced by phytohormones and a wide range of environmental cues, including water availability, nutrients, and abiotic stress, such as hypoxia and heavy metal stress [17–19]. Meanwhile, ample evidence confirmed that the formation of LR not only acts as a physical support, but also contributes to water and nutrient uptake for plant growth and development [19–21]. Different environmental clues could trigger several specific stress-induced morphogenic response (SIMR) phenotypes, including the promotion of LR formation and an inhibition of root elongation [17].. The regulation of LR formation is also tightly controlled by phytohormones, such as auxin, and the activation of cell cycle regulatory genes in response to auxin was suggested [19, 22]. Meanwhile, the involvement of some small molecules in auxin-triggered root organogenesis was confirmed in cucumber, tomato, soybean, and rapeseed plants [23–27]. These small molecules include hydrogen peroxide (H_2_O_2_), nitric oxide (NO), carbon monoxide (CO), and hydrogen gas (H_2_).

Among these, NO, a free radical gas, has been shown to have multiple physiological functions in plants [28, 29]. Besides the enhancement of plant adaptation against stresses, the functions of NO include the promotion of root hair development, adventitious rooting, and lateral root formation [30–33], although the enzymatic resource(s) of NO biosynthesis in those aforesaid processes remains elusive. In animals, the synthesis of NO from l-arginine is catalyzed by the heme-containing enzyme nitric oxide synthase (NOS) [34]. Although gene(s) encoding NOS enzymes has not been identified in plants, the mammalian NOS-like activity is detected widely [35, 36], and the inhibitors of mammalian NOS, such as *N*^G^-nitro-l-arginine methyl ester hydrochloride (l-NAME), can inhibit NO generation in plants [25, 33, 36–39]. Importantly, ample genetic evidence revealed that NO can be produced by nitrate reductase (NR), a well-known enzyme responsible for nitrogen metabolism in plants [28]. The involvement of NR-mediated NO production in stomatal closure and cold acclimation has been demonstrated genetically [37, 38]. Our previous study showed that NR-dependent NO synthesis is involved in auxin-induced hydrogen gas-mediated lateral root formation [39].

Until now, different responses in LR formation, promotion or inhibition, were respectively reported in various plant species when supplemented with nanomaterials, including MWCNTs [40–43], gold nanoparticles (Au NP, [44]), zinc oxide nanoparticles (ZnO NP [45, 46];), titanium dioxide nanoparticles (TiO_2_ NP [46];), and graphene oxide (GO [47–49];) (Table 1), and no study has yet provided definitive proof of a role of NO in above responses. In this study, the detection of endogenous NO by Greiss reagent method, laser scanning confocal microscopy (LSCM), and electron paramagnetic resonance (EPR) analyses revealed that the NO level was increased in MWCNT-treated tomato seedlings. Afterwards, LR formation was observed. We further study the involvement of NO in LR formation triggered by MWCNTs, by manipulating endogenous NO levels using NO scavenger and antagonists that inhibit NR and mammalian-like NOS activity. Further experiment revealed that NR-dependent NO might be, at least partially, essential for LR formation in response to MWCNTs. This work thus opens a new window for understanding the biological effects of nanomaterials in plants.

**Table 1:** Different responses in LR formation triggered by nanomaterials

Materials	OD (nm)	ID (nm)	Length (μm)	Species	Concentration	Effect on LR formation	Article(s)
MWCNTs	6–12	2.5–5	1–9	*Solanum lycopersicum*	5000 mg/L	Promotion	This study
MWCNTs	20–70	5–10	> 2	*Glycine max*	1000 mg/L	Inhibition	[40]
MWCNTs	6–13	2–6	2.5–20	*Lupinus elegans*; *Eysenhardtia polystachya*	10–50 μg/mL	Promotion	[41]
MWCNTs	About 9.5	–	< 1	*Lactuca sativa*	5–20 mg/L	Promotion	[42]
MWCNTs	30–40	–	–	*Arabidopsis thaliana*	50 mg/L	Promotion	[43]
Au NP	20–50	–	–	*Gloriosa superba*	500–1000 μM	Promotion	[44]
ZnO NP	< 100	–	< 1	*Triticum aestivum*	125–500 mg/L	Promotion	[45]
ZnO NP	< 50	–	–	*Cicer arietinum*	100–1000 ppm	Inhibition	[46]
TiO_2_ NP	< 50	–	–	*Cicer arietinum*	100–1000 ppm	Promotion	[46]
GO	50–200	–	–	*Oryza sativa; Malus domestica*	0.01–1 mg/L, 5–50 mg/L, 0.1–10 mg/L	Promotion Inhibition	[47–49]

*Au NP*, gold nanoparticles; *ZnO NP*, zinc oxide nanoparticles; *TiO*_*2*_ *NP*, titanium dioxide nanoparticles; *GO*, graphene oxide

## Materials and Methods

### Chemicals

Unless stated otherwise, all the other chemicals were obtained from Sigma-Aldrich (St Louis, MO, USA). MWCNTs, purchased from Sigma-Aldrich, was characterized as previously described [50]. The outer diameter, inter diameter, and the length of MWCNTs were 6–12 nm, 2.5–5 nm, and 1–9 μm, respectively. After sonication treatment, the obtained homogenate colloidal suspension was sterilized and used.

Other carbon nanoparticles were obtained from Nanjing XFNANO Materials Tech Co., Ltd., including single-walled carbon nanotubes (SWCNTs, XFS22; purity > 95%, diameter 1–2 nm, length 5–30 μm, special surface area > 1075 m^2^/g), graphene (XF001W; purity ~ 99%, diameter 0.5–5 μm, thickness ~ 0.8 nm, single layer ratio ~ 80%, BET surface area 500~1000 m^2^/g; electrical resistivity ≦ 0.30 Ω.cm), and active carbon (AC, XFP06; purity > 95%, particle size 5 ± 1 μm, pore volume 1–1.2 cm^3^/g, aperture 2.0–2.2 nm, special surface area ~ 1500–1700 m^2^/g).

Additionally, sodium nitroprusside (SNP) was used as a NO-releasing compound [30–33]. 2-(4-Carboxyphenyl)-4,4,5,5-tetramethylimidazoline-1-oxyl-3-oxide potassium salt (cPTIO) was regarded as a scavenger of NO [51–54]. Tungstate (Tg; an inhibitor of NR [28, 33, 37, 55–57];) and *N*^G^-nitro-L-arginine methyl ester hydrochloride (NAME; an inhibitor of mammalian NOS-like enzyme [25, 33, 36–39];) were also applied. In this study, the concentrations of above chemicals were determined in the pilot experiments, from which the significant responses were observed.

### Plant Material and Growth Conditions and Determination of LR Formation

Tomato (*Solanum lycopersicum* L.) seeds “Jiangshu 14” were kindly supplied by Jiangsu Agricultural Institutes, Nanjing, Jiangsu Province, China. Selected seeds of identical size were germinated in distilled water at 25 ± 1 °C in the dark for 3 days. The selected identical seedlings with radicles 2–3 mm were then transferred to 6 mL treatment solutions containing the indicated concentrations of MWCNTs, 200 nM 1-naphthylacetic acid (NAA; a well-known auxin), 0.1 mM SNP, 0.2 mM cPTIO, 20 μM tungstate (Tg), 0.2 mM NAME, and other carbon nanoparticles, including 5 mg/mL single-walled carbon nanotubes (SWCNTs), graphene, and active carbon (AC), alone or in combination for the indicated time points. Seedlings were grown in an illuminating incubator (25 ± 1 °C) with a light intensity of 200 μmol m^−2^ s^−1^ at 14/10 h (light/dark) photoperiod.

After treatments, pictures were taken, and the number and length of emerged lateral root (> 1 mm) per seedling were then determined by using the Image J software (http://rsb.info.nih.gov/ij/) [39, 58]. As described previously, only the lateral root-inducible segments were used for the subsequent analysis.

### Imaging of MWCNT Distribution by Transmission Electron Microscopy

The distribution of MWCNTs in tomato seedling root was characterized using the transmission electron microscopy (TEM; JEOL, JEM-200CX, Tokyo, Japan). Sample preparation for TEM analysis was according to the previous protocol [59].

### Imaging of Endogenous NO by Laser Scanning Confocal Microscope

NO imaging was carried out by using a fairly specific NO fluorescent probe 4-amino-5-methylamino-2′,7′-difluorofluorescein diacetate (DAF-FM DA). After the probe was thoroughly washed, the images were obtained using the Zeiss LSM 710 confocal microscope (Carl Zeiss, Oberkochen, Germany, excitation at 488 nm, emission at 500–530 nm for NO analysis). In our experiment, 20 individual samples were randomly selected and measured per treatment. Photographs are representative of identical results.

### NO Content Determined by Griess Reagent Assay

According to the methods previously described [50], NO content was determined with the Griess reagent assay. Importantly, for escaping the interfering caused by the concentrated nitrate and nitrite contents in plants, the identical samples preincubated in 200 μM cPTIO (the scavenger of NO) for 30 min were regarded as the blank samples. After the addition of Griess reagent for 30 min, absorbance was recorded at 540 nm, and NO content was determined by comparison to a standard curve of NaNO_2_.

### Determination of NO with Electron Paramagnetic Resonance (EPR)

According to our previous methods [39, 55, 60], the determination of NO level using electron paramagnetic resonance (EPR) was carried out. The organic solvent layer was used to determine NO on a Bruker A300 spectrometer (Bruker Instrument, Karlsruhe, Germany) under the following conditions: room temperature; microwave frequency, 9.85 GHz; microwave power, 63.49 mW; and modulation frequency, 100.00 kHz.

### Determination of Nitrate Reductase (NR) Activity

The NR activity was detected spectrophotometrically at 540 nm according to the previous method [57]. The produced nitrite was determined spectrophotometrically at 540 nm by the addition of 1 mL of 1% (w/v) sulfenilamide in 3 M HCl together with 1 mL of 0.02% (v/v) n-(1-naphthyl)-ethylenediamine.

### Statistical Analysis

Where indicated, results were expressed as the mean values ± SE of three independent experiments with three biological replicates for each. Statistical analysis was performed using the SPSS Statistics 17.0 software. For statistical analysis, Duncan’s multiple test (*p* < 0.05) was chosen as appropriate.

## Results

### MWCNTs not only Entry into Root Cells, but also Promote LR Formation

**Fig. 1:**
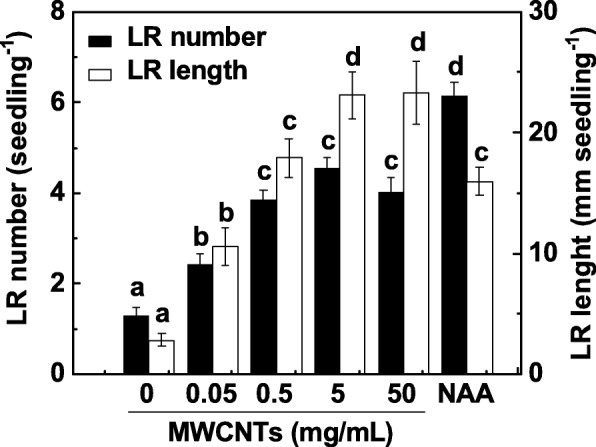
MWCNT-induced tomato LR formation was in an approximately dose-dependent manner. Three-day-old tomato seedlings were treated with 200 nM NAA and the indicated concentrations of MWCNTs, respectively. The number and length of emerged lateral root (> 1 mm) per seedling were then determined after 3 days of treatment. There were 30 (10 × 3) plants in three biological replicates, and the experiments were conducted for 3 times. Data are the means ± SE. Within each set of experiments, bars denoted by the same letter did not differ significantly at *p* < 0.05 level according to Duncan’s multiple test

LR formation is a major determinant of root systems architecture. To investigate the effect of MWCNTs on LR formation, 3-day-old tomato seedlings were incubated with a range of concentrations of MWCNTs (0.05, 0.5, 5, and 50 mg/mL) for 3 days. The application of 1-naphthylacetic acid (NAA) was regarded as a positive control. In our experiment, both LR number and length were determined as two parameters of LR formation. As shown in Fig. 1, compared to the control samples, exogenous MWCNTs significantly induced LR formation in an approximately dose-dependent manner, with a maximal effect in 5 and 50 mg/mL. Similar inducible response was observed when 200 nM NAA was administrated. Considering the cost of MWCNTs and inducible response in LR formation, 5 mg/mL MWCNTs was applied in the following experiments.

**Fig. 2:**
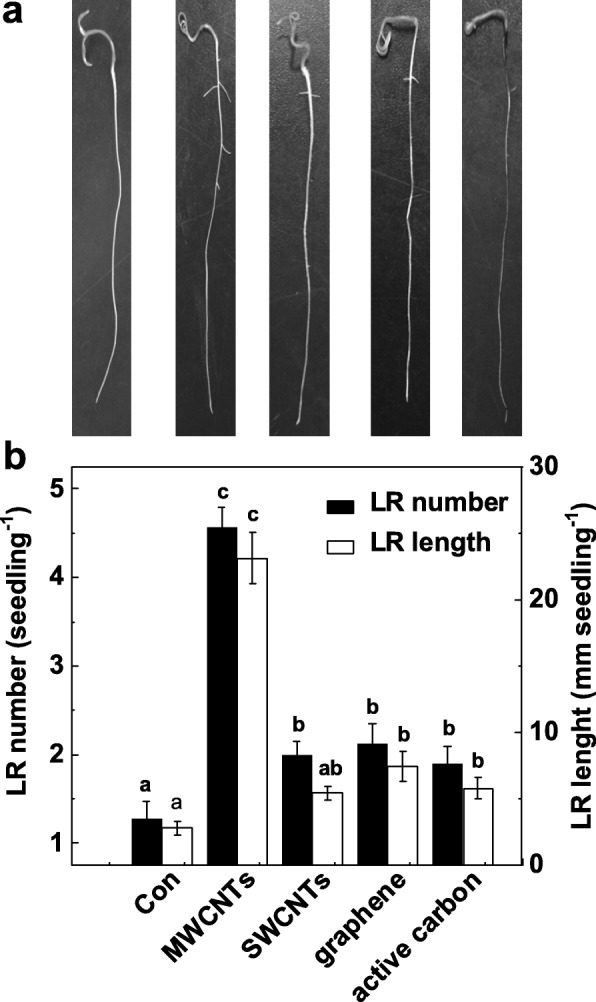
Changes in lateral root formation in response to different carbon nanoparticles. Three-day-old tomato seedlings were treated with distilled water (Con), 5 mg/mL MWCNT, single-walled carbon nanotubes (SWCNTs), graphene, and active carbon (AC), respectively, for another 3 days. **a** Representative photos were then taken. **b** The number and length of emerged lateral root (> 1 mm) per seedling were then determined as well. Scale bar = 50 mm. There were 30 (10 × 3) plants in three biological replicates, and the experiments were conducted for 3 times. Data are the means ± SE. Within each set of experiments, bars denoted by the same letter did not differ significantly at *p* < 0.05 level according to Duncan’s multiple test

To validate the specific function of MWCNTs in the induction of LR formation, we further investigate whether the other allotropies of MWCNTs also have such inducible effects. As shown in Fig. 2a, all these carbon nanomaterials exhibited toxic effects on shoot growth (data not shown). Interestingly, the application of MWCNTs, single-walled carbon nanotubes (SWCNTs), graphene, and active carbon with identical concentration (5 mg/mL) could differentially result in the increases in LR number and length, compared to the chemical-free control plants (Fig. 2b). Among these chemicals, the maximal inducible response was discovered in MWCNT-incubated tomato seedlings.

**Fig. 3:**
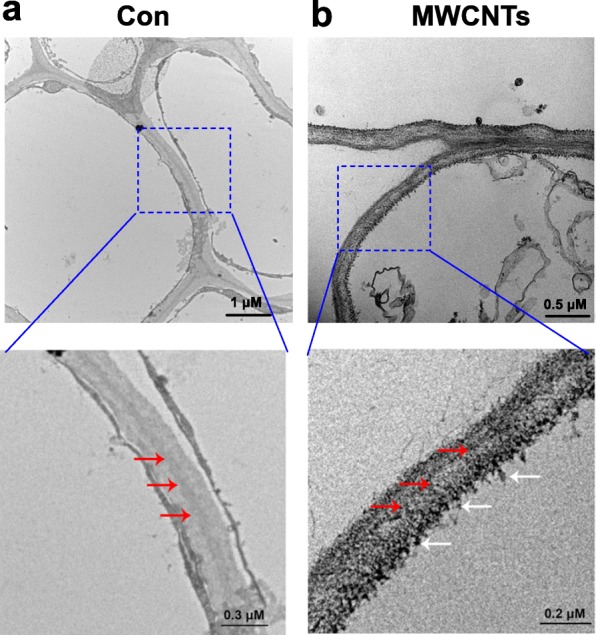
Distribution of MWCNTs in tomato roots. TEM images of 3-day-old tomato seedlings treated with distilled water (Con; **a**) or 5 mg/mL MWCNTs (**b**) for 1 day were taken. Red arrow indicates cell wall, while white arrow indicates MWCNTs

By the aid of transmission electron microscopy (TEM), the distribution of MWCNTs can be evaluated easily. The results shown in Fig. 3 revealed that MWCNTs, when exogenously applied, could be absorbed by tomato seedlings, and the distribution of MWCNTs was found to be in root cells. This result can be understood, since seedling roots are directly cultured in liquid solution containing MWCNTs.

### MWCNT-Induced NO Synthesis and Thereafter LR Formation Were Sensitive to cPTIO, a Scavenger of NO

**Fig. 4:**
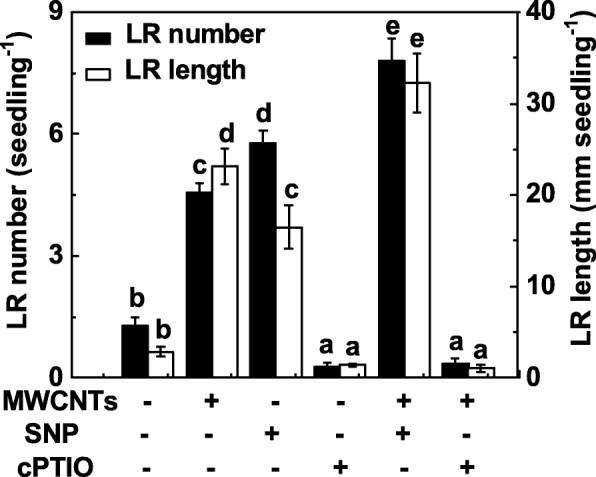
MWCNT-induced LR formation was sensitive to the removal of endogenous NO with cPTIO, its scavenger. Three-day-old tomato seedlings were treated with distilled water, 5 mg/mL MWCNT, 0.1 mM SNP, 0.2 mM cPTIO, alone or in combination for 3 days. Afterwards, the number and length of emerged lateral root (> 1 mm) per seedling were then determined. There were 30 (10 × 3) plants in three biological replicates, and the experiments were conducted for 3 times. Data are the means ± SE. Within each set of experiments, bars denoted by the same letter did not differ significantly at *p* < 0.05 level according to Duncan’s multiple test

To investigate whether NO is also involved in MWCNT-induced LR formation, the function of NO in LR formation elicited by MWCNTs was assessed by manipulating endogenous NO levels using NO-releasing compound and the scavenger. Similar to the previous results [31], the administration of sodium nitroprusside (SNP) could result in the induction of LR formation, and an additive response was observed when SNP and MWCNTs were applied together (Fig. 4). When 2-(4-carboxyphenyl)-4,4,5,5-tetramethylimidazoline-1-oxyl-3-oxide potassium salt (cPTIO; a scavenger of NO) was added, the promotion responses in LR formation caused by MWCNTs were significantly impaired. Alone, cPTIO could inhibit LR development, compared to the chemical-free control, indicating the important role of endogenous NO in root organogenesis.

**Fig. 5:**
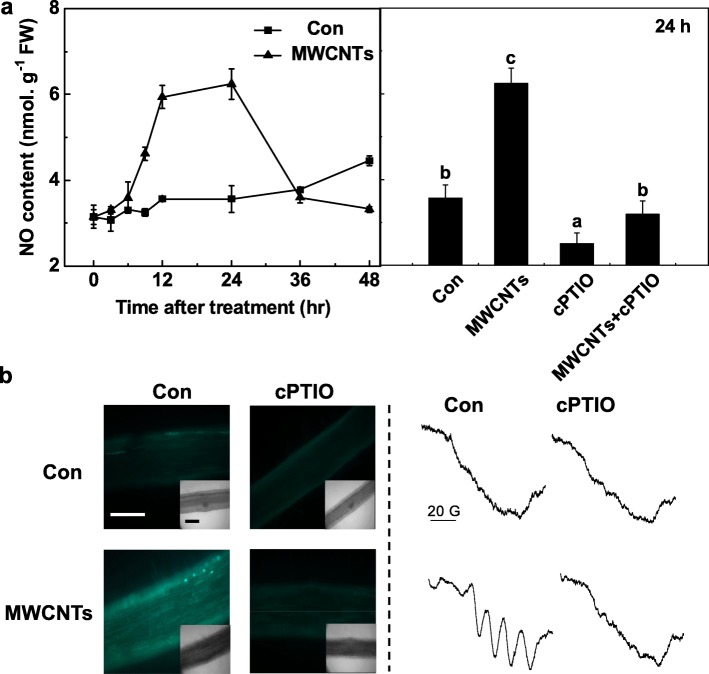
MWCNT-induced NO production was blocked by cPTIO, the scavenger of NO. Three-day-old tomato seedlings were treated with distilled water and 5 mg/mL MWCNTs with or without 0.2 mM cPTIO, respectively. **a** Changes in NR activity (left), and NO production (right) determined using Greiss reagent method. **b** After treamtent for 24 h, the NO signal was analyzed by LSCM (left) and EPR (right). Scale bar = 0.1 mm. Data are the means ± SE. Bars denoted by the same letter did not differ significantly at *p* < 0.05 level according to Ducan’s multiple test

In order to further evaluate the important role of endogenous NO in MWCNT response, a time course of NO production in vivo was firstly detected with Greiss reagent method. During above determination, the identical filtrate pretreated with cPTIO was regarded as a blank for the accurate results. It was observed that NO production in tomato seedling roots was increased dramatically till 24 h after MWCNT treatment and then recovers to the initial levels (48 h; Fig. 5a). Above maximal level of endogenous NO triggered by MWCNTs for 24 h was obviously abolished by cPTIO, a scavenger of NO, suggesting the specific role of NO.

To confirm above results, both LSCM and ESR were adopted. Firstly, the changes in endogenous NO levels in seedling roots of tomato were monitored by labeling NO using the cell-permeable, fairly NO-specific fluorescent probe DAF-FM DA and imaging with LSCM. Similar to the previous results (Fig. 5a), in the presence of cPTIO, the increased DAF-FM-dependent fluorescence intensity triggered by MWCNTs was greatly abolished (Fig. 5b). These results implied that the DAF-FM-triggered fluorescence is related to endogenous NO levels in tomato seedling roots.

MWCNT-induced NO production was confirmed by EPR spectroscopy. As expected, seedling roots treated for 24 h with MWCNTs presented the typical hyperfine structure triplet of the NO complex. However, the addition of cPTIO abolished above signal, indicating that MWCNT exposure did result in a strong NO production (Fig. 5b). Collectively, these data suggested that NO synthesis might be required for MWCNT-triggered LR formation in tomato seedlings.

### NR Might Be Responsible for MWCNT-Induced NO Production and Thereafter LR Formation

**Fig. 6:**
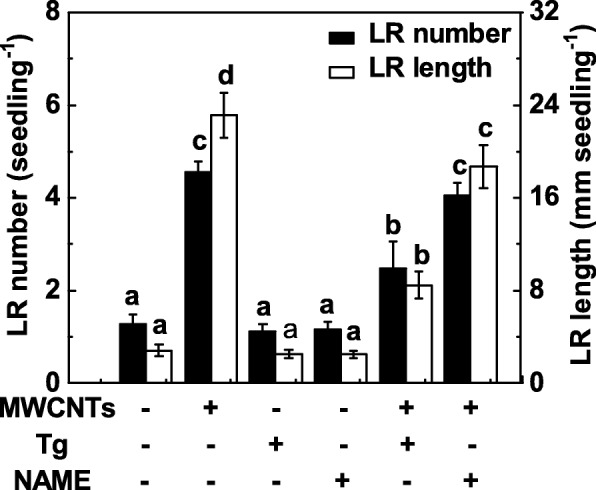
Changes in LR formation in response to MWCNTs and two inhibitor of NO synthesis. Three-day-old tomato seedlings were treated with 5 mg/mL MWCNT, 20 μM tungstate (Tg), 0.2 mM NAME, alone or in combination for 3 days. Afterwards, the number and length of emerged lateral root (> 1 mm) per seedling were then determined. There were 30 (10 × 3) plants in three biological replicates, and the experiments were conducted for 3 times. Data are the means ± SE. Within each set of experiments, bars denoted by the same letter did not differ significantly at *p* < 0.05 level according to Duncan’s multiple test

Since NR and mammalian-like NOS are two major enzymes related to NO synthesis in plants, both tungstate (a NR inhibitor) and NAME (a mammalian NOS inhibitor) were applied in the subsequent experiment. Here, tungstate treatment substantially suppressed the promotion of LR formation in MWCNT-treated tomato seedling roots (Fig. 6). Comparatively, the induction of LR formation triggered by MWCNTs was not strongly inhibited by the addition of NAME, indicating that mammalian-like NOS might be not the target NO synthetic enzyme responsible for NO production elicited by MWCNTs. It was also observed that a slight but no significant decrease in LR formation was observed in tomato seedlings when either tungstate or NAME was separately applied.

**Fig. 7:**
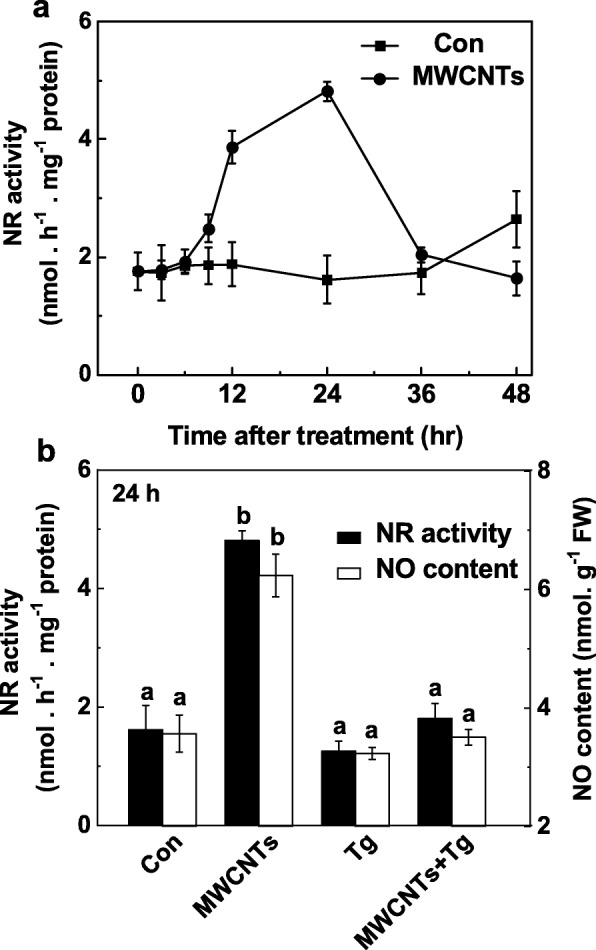
MWCNT-induced NO production was blocked by tungstate, an inhibitor of NR. Three-day-old tomato seedlings were treated with distilled water and 5 mg/mL MWCNTs with or without 20 μM tungstate (Tg). Changes in NR activity (**a**) and NO production (**b**) determined using Greiss reagent method. Data are the means ± SE. Bars denoted by the same letter did not differ significantly at *p* < 0.05 level according to Ducan’s multiple test

The role of NR in MWCNT-triggered LR formation was further examined by monitoring NO production in response to applied MWCNTs with or without tungstate. Compared to the changes in endogenous NO production (Fig. 5a), time-course analysis in NR activity showed the similar tendency (Fig. 7a), also peaking at 24 h after treatment with MWCNTs. These results suggested that MWCNT-induced increase in NO production may mainly result from enhanced activity of NR. Consistently, the inhibition of NR-dependent NO production by tungstate was confirmed by using Greiss reagent method (Fig. 7b), LSCM, and EPR (Additional file 1: Figure S1).

## Discussion

Phytotoxicity is a significant consideration in understanding the potential environmental impact of nanoparticles [4, 7, 61–63]. Abundant evidence revealed that MWCNTs are toxic to plants, including inducing oxidative damage, inhibiting seed germination, root growth, and development [11, 63, 64]. However, being as a phenotype of SIMR, root branching through lateral root formation is an important component of the adaptability of the root system to various environmental cues [17]. In this work, we integrated biological, pharmacological, and biochemical analysis to show the involvement of NR-mediated NO production in MWCNT-induced LR formation, at least partially in our experimental conditions. Also, the function of NO in root organogenesis stimulated by MWCNTs emphasized the central roles of this second messenger involved in plant developmental process and adaption against stress [29–33, 37, 38].

First, we confirmed that 5 mg/mL MWCNTs (OD 6–12 nm) could enter into root tissues (Fig. 3). Afterwards, the induction of tomato LR formation was observed (Fig. 1), mimicking the induction roles of NAA and SNP (Fig. 4), a well-known NO-releasing compound [30, 31]. Similar inducing responses were discovered in resinous trees [41], lettuce [42], and Arabidopsis [43] when challenged with MWCNTs (OD 6–13 nm, about 9.5 nm, and 30–40 nm, respectively). For example, the application with either pristine MWCNT (p-MWCNT) or carboxyl-functionalized MWCNT (c-MWCNT) (average diameter 9.5 nm) could promote the development of LR in lettuce seedlings [42]. By contrast, the inhibition of primary root and even LR formation were simultaneously found in soybean plants when subjected to MWCNTs (OD 20–70 nm [40];). By comparing with the data in outer diameter of MWCNTs (Table 1), we supposed that MWCNT-exhibited effects on LR formation varied with their diameters, showing the promotion with lower diameter and the inhibition with higher diameter. Certainly, related mechanism should be carefully investigated. Similar phenomenon was confirmed in plant salinity tolerance [50]. Combined with above results, it was further deduced that the function of nanomaterials may vary from species, and vice versa, different types of nanomaterials may cause various biological effects. However, other influencing factors, such as different doses of MWCNTs [48] and even plant growth conditions, could be not easily ruled out.

Compared with other nanomaterials, including SWCNT, graphene, and AC with an identical concentration, the maximal induction in LR formation and even toxic effects on shoot growth were observed in MWCNTs (Fig. 2). These might be related to the special physical characteristics of MWCNTs, one type of nanomaterials that have high electrical conductivity, large specific surface area, high aspect ratio, and remarkable thermal stability [65]. The toxic effects of nanomaterials have been widely reported in cucumber, cabbage, carrot, onion etc. [66, 67].

Ample evidence showed that NO, acting as a signaling molecule, can regulate a wide range of plant processes from environmental adaptation to development and the latter of which includes seed germination and root organogenesis [29, 68–73]. Our subsequent experiment revealed that NO may be involved in MWCNT-induced LR formation. Although several methods for imaging NO production in plant cells have been applied, the disadvantages, including the lack of sensitivity and the interference by NO-independent molecules, may exist in each method [74]. Thus, three methods responsible for NO imaging and determination, including Greiss reagent method, LSCM, and EPR, together with the application of cPTIO, a scavenger of NO, were applied in our experimental conditions. By using three methods, we observed that an increased endogenous NO production induced by MWCNTs in tomato seedlings was abolished by cPTIO (Fig. 5), a scavenger of NO [30–32]. Importantly, this process was correlated to the biological response of MWCNT-induced LR development, which was severely blocked when cPTIO was applied simultaneously (Fig. 4).

Further evaluation of these responses and the potential source(s) of NO induced by exogenously applied MWCNTs revealed that NO production and thereafter LR formation could be attributed to NR activity. In plants, NO production mainly generates from NR and mammalian NOS-like protein [28]. However, plant NOS gene is still not identified [35, 75, 76], although some experiments using the inhibitors of the mammalian NOS enzyme provided some evidence of l-arginine-dependent pathway in NO production [36, 76]. NR is confirmed to be the most important sources of NO in plants [28]. Previous studies showed that NR-dependent NO production functions as a nitrate-related signal involved in the regulation of root architecture [32, 33]. Besides, NR-dependent NO production was closely associated with in cold acclimation [38], salinity tolerance [50], and abscisic acid-induced stomatal closure [77]. Our results further revealed that tungstate (an inhibitor of NR) obviously impaired MWCNT-induced LR formation, especially in LR length (Fig. 6). By contrast, there was only a slight decrease in LR length, and no significant difference observed in LR number when l-NAME (an inhibitor of mammalian NOS) was used. Consistently, biochemical assay showed that NR activity was increased obviously by MWCNTs (Fig. 7a), paralleled to the changes in NO production (Fig. 5a). Above responses could be totally blocked by tungstate (Fig. 7a, Additional file 1: Figure S1). We thus deduced that the increased endogenous NO production induced by MWCNTs was mainly attribute to NR pathway. Certainly, further genetic evidence should be investigated.

## Conclusion

**Fig. 8:**
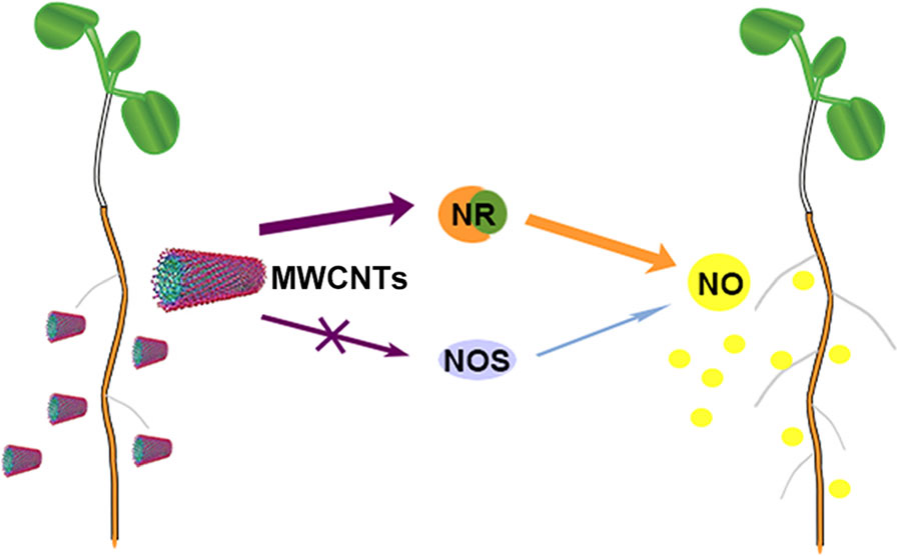
Schematic representation of the proposed MWCNT-induced tomato lateral root formation mainly via NR-dependent NO production. The role of mammalian NOS-like enzyme was preliminarily ruled out

In summary, we provide evidence to show that MWCNT-induced NO production via NR might be required for tomato lateral root formation and this was summarized in Fig. 8. Importantly, above findings provide insights into the intricate molecular mechanism of MWCNTs functions in plants.

## Supplementary information

**Additional file 1: Figure S1.** Tg inhibits MWCNTs-induced NO accumulation. 3-day-old tomato seedlings were treated with distilled water and 5 mg/mL MWCNTs with or without 20 μM tungstate (Tg). The NO signal was analyzed by LSCM (A) and EPR (B) after treated for 24 h. Scale bar = 0.1 mm.

## Data Availability

All data are fully available without restriction.

## Abbreviations

CO: Carbon monoxide
cPTIO: 2-(4-Carboxyphenyl)-4,4,5,5-tetramethylimidazoline-1-oxyl-3-oxide potassium salt
DAF-FM DA: 4-Amino-5-methylamino-2′,7′-difluorofluorescein diacetate
EPR: Electron paramagnetic resonance
GO: Graphene oxide
H_2_: Hydrogen gas
H_2_O_2_: Hydrogen peroxide
l-NAME: *N*^G^-Nitro-l-arginine methyl ester
LR: Lateral root
LSCM: Laser scanning confocal microscopy
MWCNTs: Multi-walled carbon nanotubes
NAA: 1-Naphthylacetic acid
NO: Nitric oxide
NOS: Nitric oxide synthase
NR: Nitrate reductase
ROS: Reactive oxygen species
SIMR: Stress-induced morphogenic response
SNP: Sodium nitroprusside
SWCNTs: Single-walled carbon nanotubes
TEM: Transmission electron microscopy
Tg: Tungstate
