# Supplementary material for: A Novel Mechanism Underlying Multi-walled Carbon Nanotube-Triggered Tomato Lateral Root Formation: the Involvement of Nitric Oxide
Source: Nanoscale Res Lett. 2020 Feb 26;15:49. doi: 10.1186/s11671-020-3276-4 (PMC7044399; doi:10.1186/s11671-020-3276-4)
Supplement: Supplementary file 1 — Additional file 1: Figure S1. Tg inhibits MWCNTs-induced NO accumulation. 3-day-old tomato seedlings were treated with distilled water and 5 mg/mL MWCNTs with or without 20 μM tungstate (Tg). The NO signal was analyzed by LSCM (A) and EPR (B) after treated for 24 h. Scale bar = 0.1 mm. [file 11671_2020_3276_MOESM1_ESM.docx]

**Fig. S1.**

**Fig. S1** Tg inhibits MWCNTs-induced NO accumulation. 3-day-old tomato seedlings were treated with distilled water and 5 mg/mL MWCNTs with or without 20 µM tungstate (Tg). The NO signal was analyzed by LSCM (A) and EPR (B) after treated for 24 h. Scale bar = 0.1 mm.

**Con**

**MWCNTs**

**Con Tg**

**a**


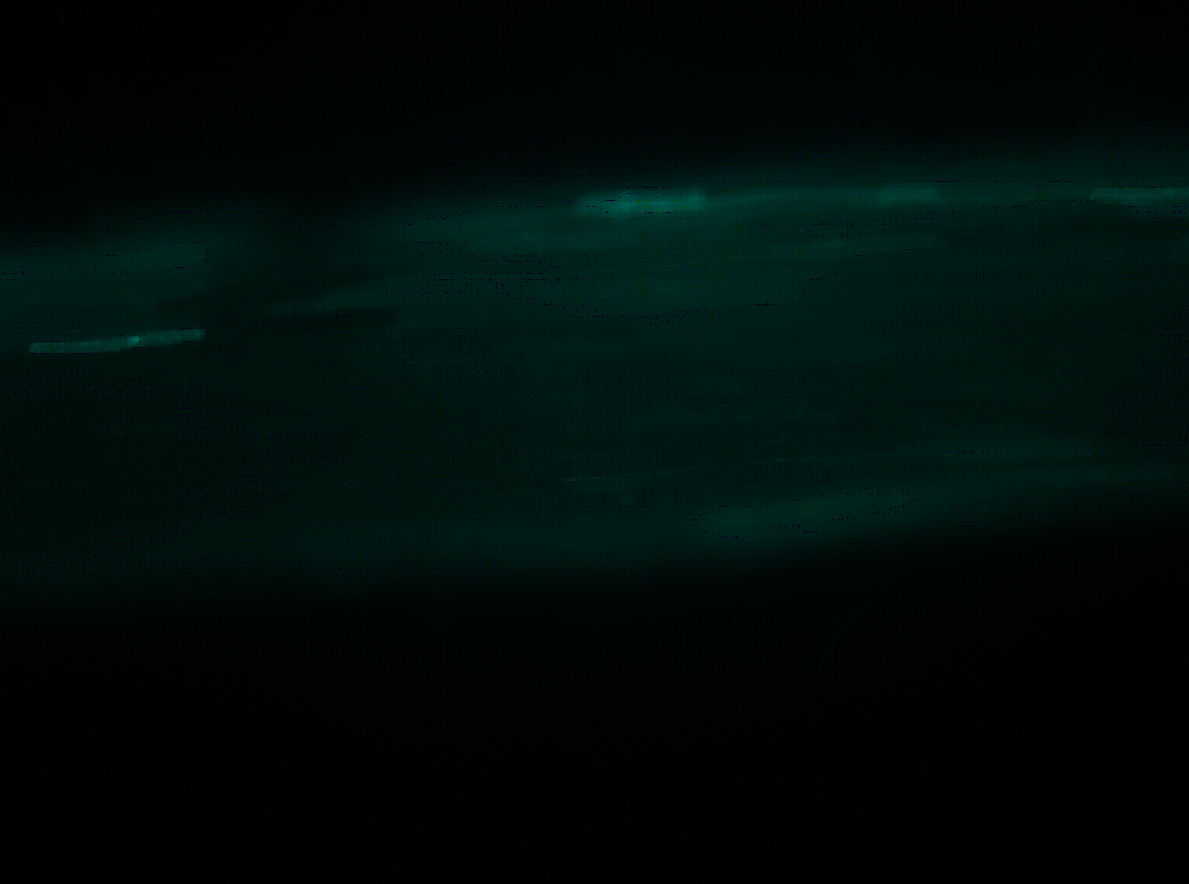

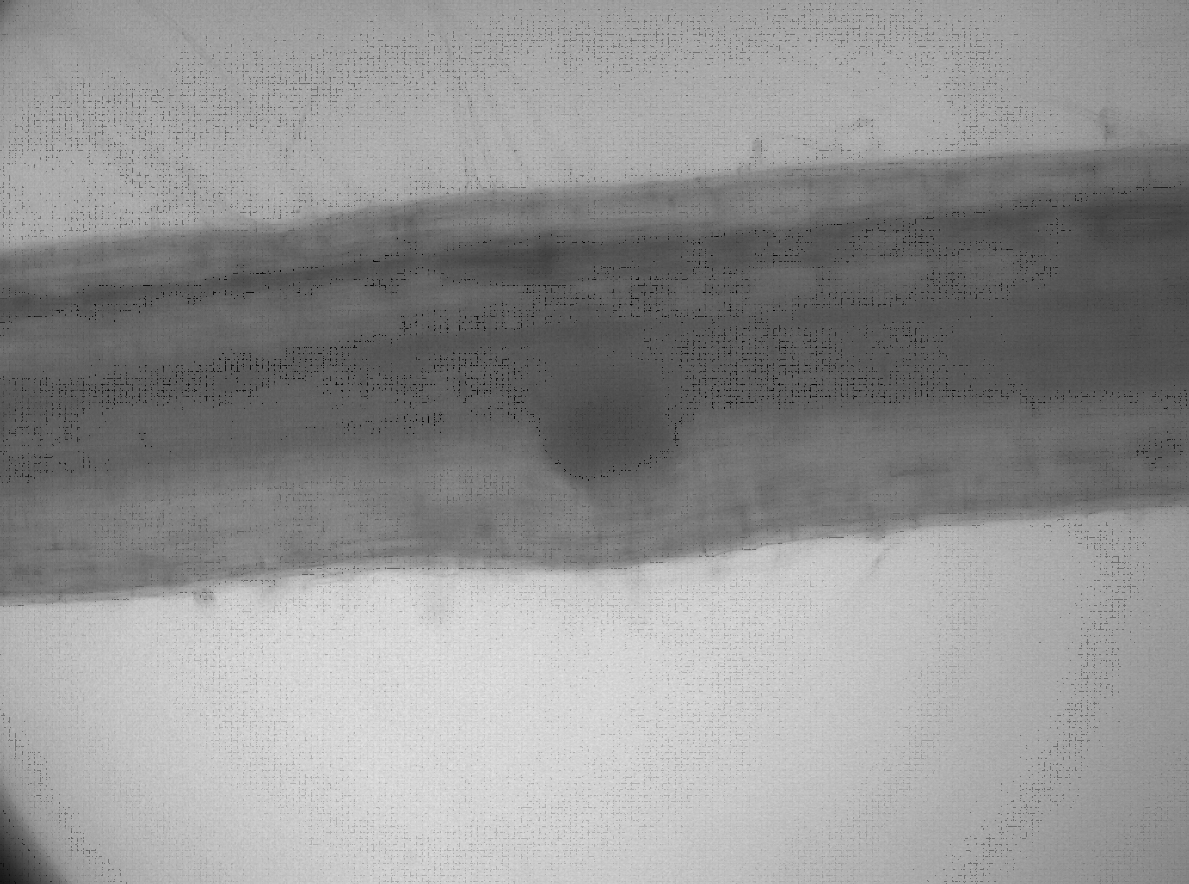

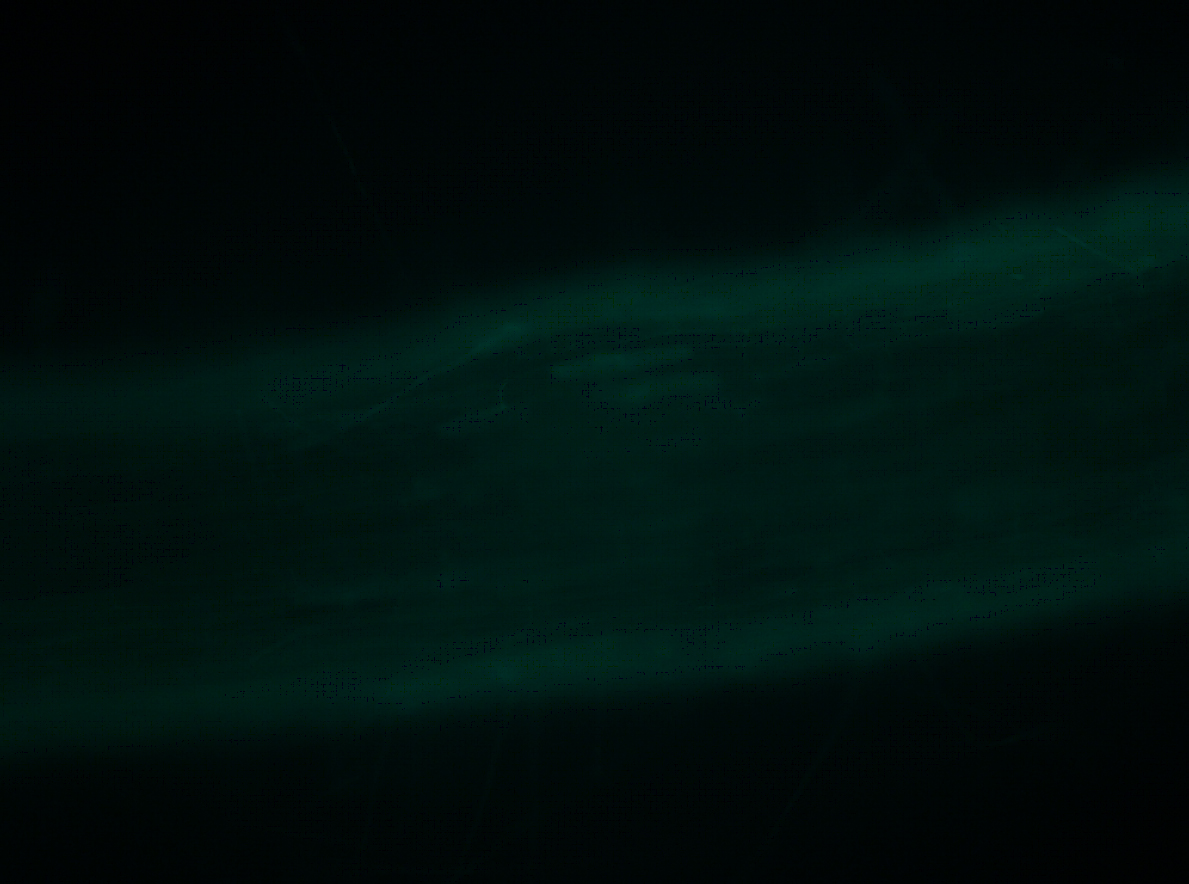

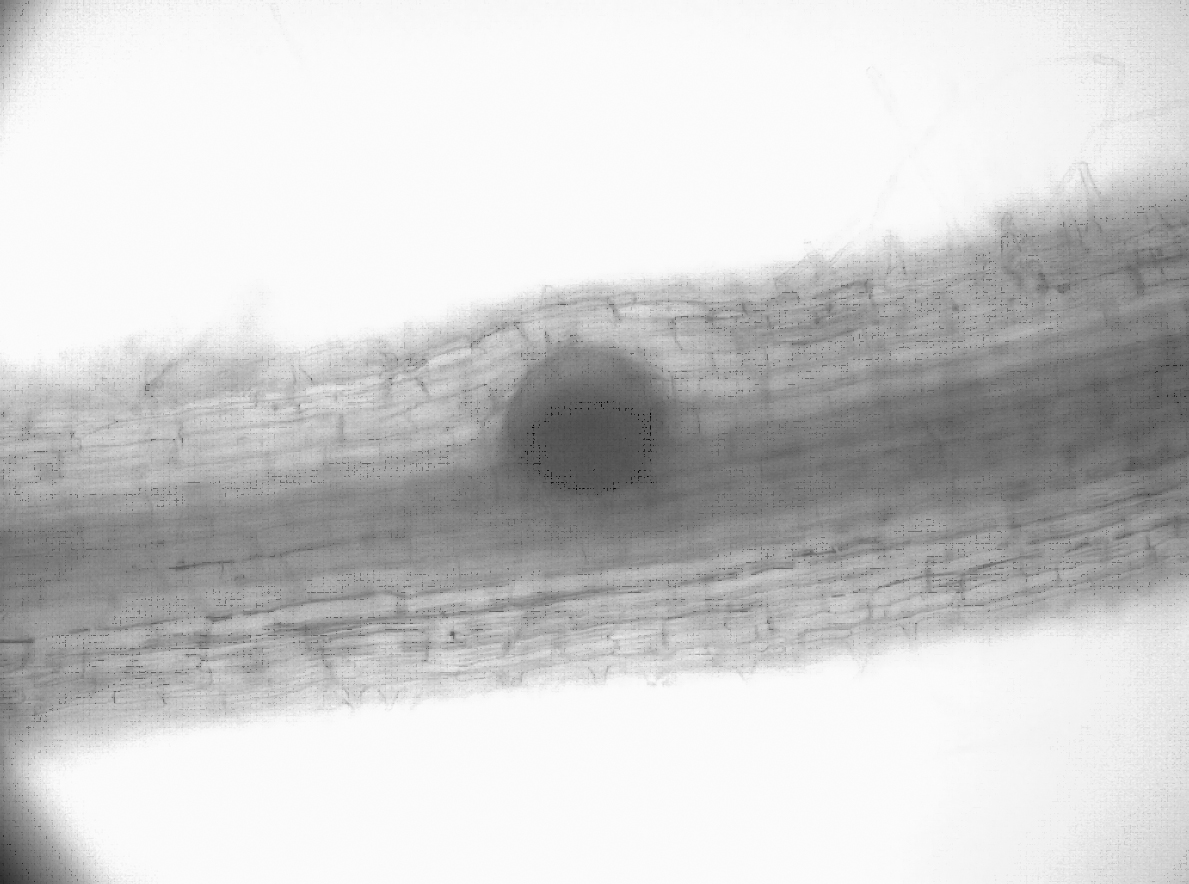

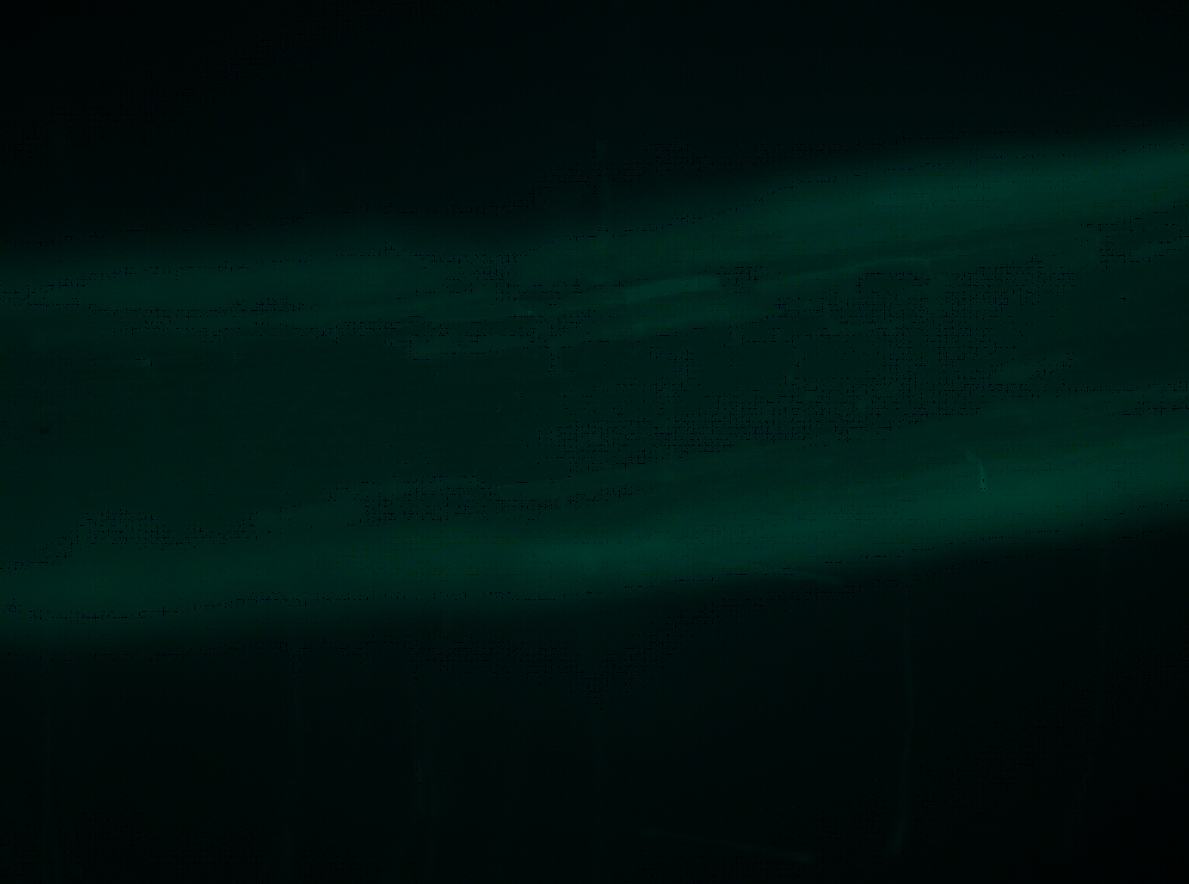

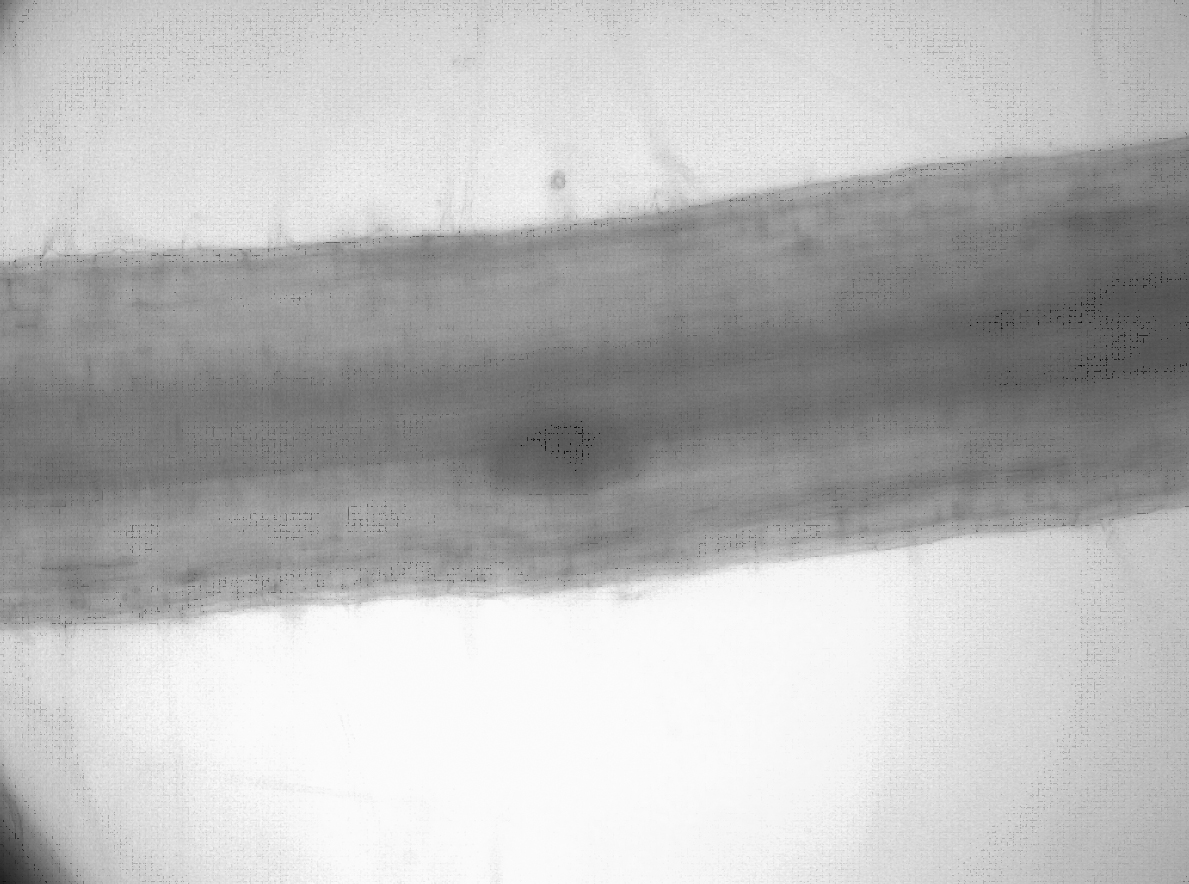

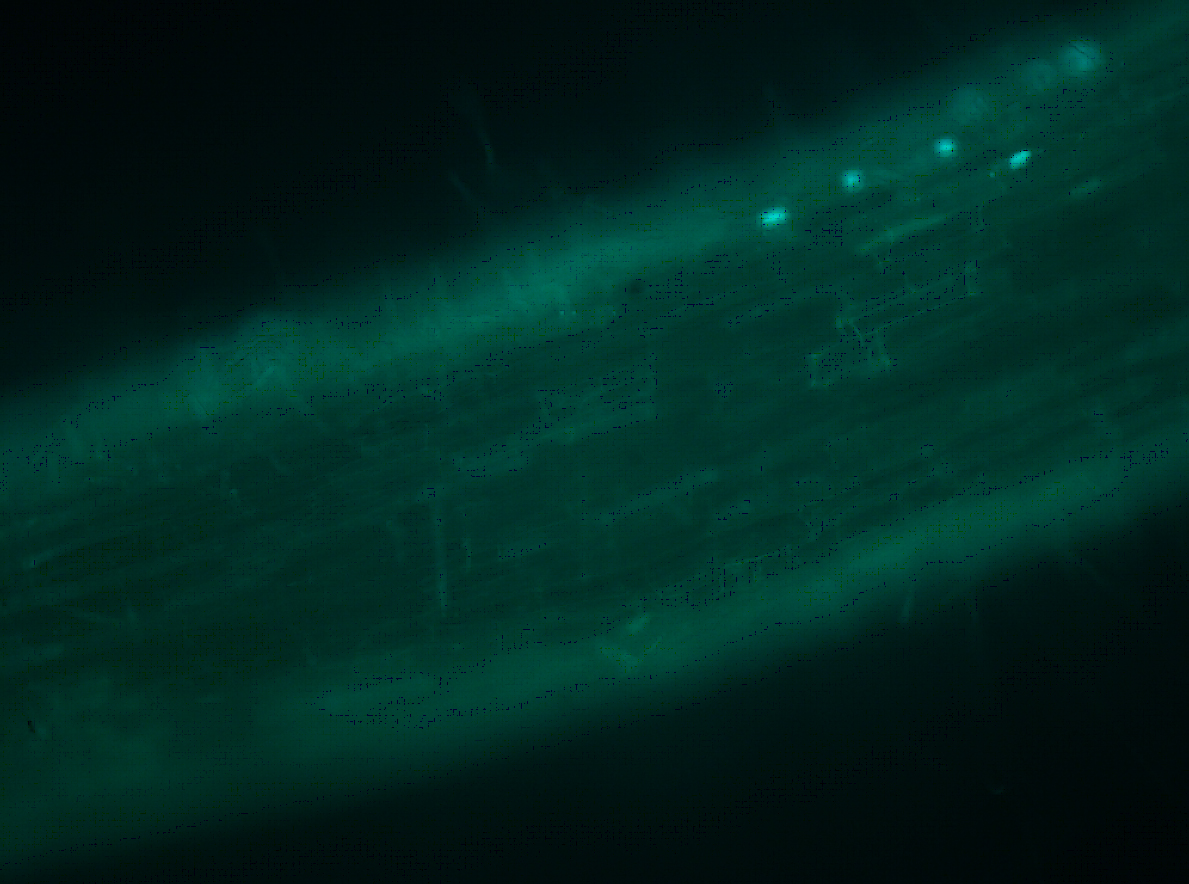

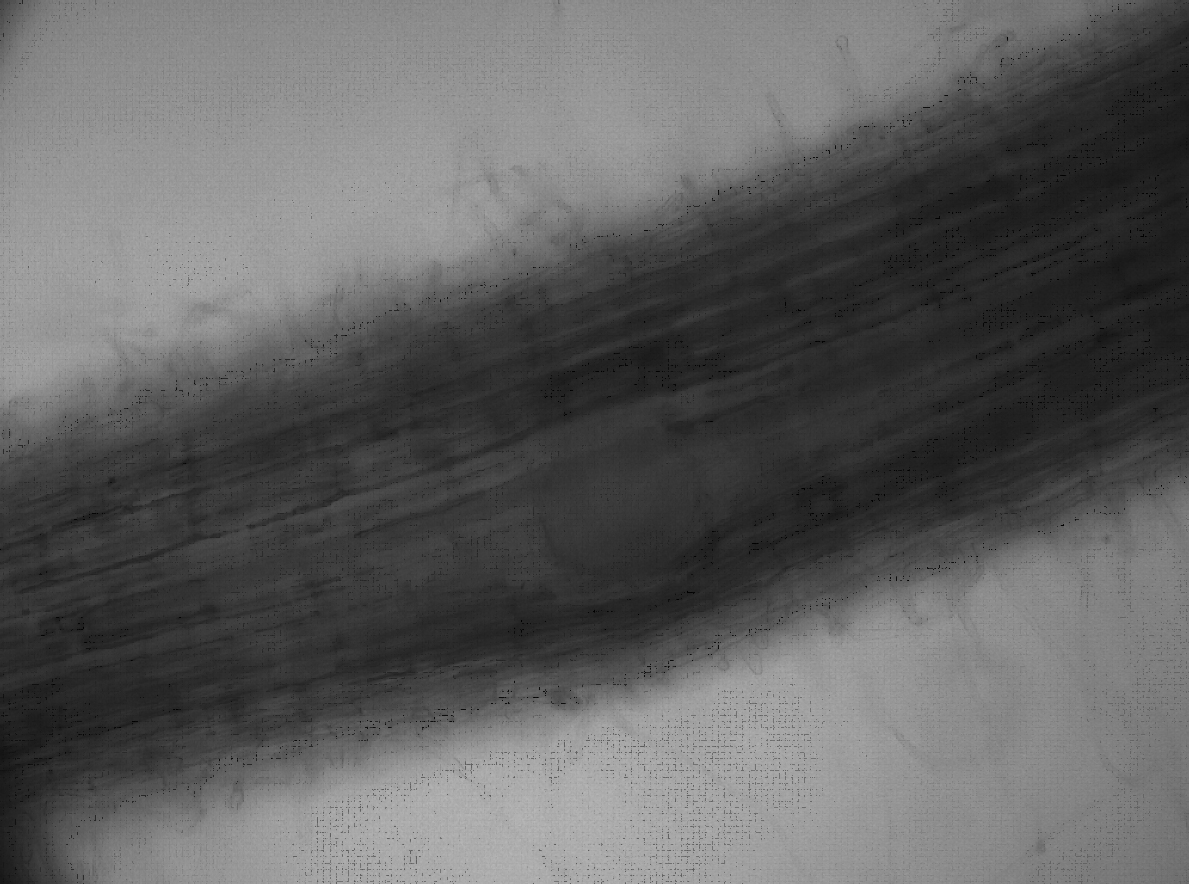


**Con Tg**

**b**
